# Multimodal Balance Training Supported by Rhythmic Auditory Stimuli in Parkinson Disease: Effects in Freezers and Nonfreezers

**DOI:** 10.1093/ptj/pzaa146

**Published:** 2020-07-31

**Authors:** Tamine T C Capato, Nienke M de Vries, Joanna IntHout, Jordache Ramjith, Egberto R Barbosa, Jorik Nonnekes, Bastiaan R Bloem

**Affiliations:** Donders Institute for Brain, Cognition and Behaviour, Department of Neurology, and Center of Expertise for Parkinson & Movement Disorders, Radboud University Medical Center; Nijmegen, the Netherlands; and Movement Disorders Clinic, Department of Neurology, University of São Paulo, São Paulo, Brazil; Donders Institute for Brain, Cognition and Behaviour, Department of Neurology, and Center of Expertise for Parkinson & Movement Disorders, Radboud University Medical Center; Department for Health Evidence, Radboud Institute for Health Sciences, Radboud University Medical Center; Department for Health Evidence, Radboud Institute for Health Sciences, Radboud University Medical Center; Movement Disorders Clinic, Department of Neurology, University of São Paulo; Donders Institute for Brain, Cognition and Behaviour, Department of Neurology, and Center of Expertise for Parkinson & Movement Disorders, Radboud University Medical Center; and Department of Rehabilitation, Sint Maartenskliniek, Nijmegen, the Netherlands; Donders Institute for Brain, Cognition and Behaviour, Department of Neurology, and Center of Expertise for Parkinson & Movement Disorders, Radboud University Medical Center

## Abstract

**Objective:**

To fulfill the potential of nonpharmacological interventions for people with Parkinson disease (PD), individually tailored treatment is needed. Multimodal balance training supported by rhythmic auditory stimuli (RAS) can improve balance and gait in people with PD. The purpose of this study was to determine whether both freezers and nonfreezers benefit.

**Methods:**

A secondary analysis was conducted on a large randomized controlled trial that included 154 patients with PD (Hoehn & Yahr Stages 1–3 while ON-medication) who were assigned randomly to 3 groups: (1) multimodal balance training with RAS delivered by a metronome (RAS-supported multimodal balance training); (2) regular multimodal balance training without rhythmic auditory cues; and (3) a control intervention (involving an educational program). Training was performed for 5 weeks, twice per week. The primary outcome was the Mini-BESTest score directly after the training period. Assessments were performed by a single, masked assessor at baseline, directly postintervention, and after 1-month and 6-month follow-up. Outcomes were analyzed in 1 analysis, and the results were presented separately for freezers and nonfreezers with a linear mixed model, adjusted for baseline Mini-BESTest scores, Unified Parkinson’s Disease Rating Scale scores, and levodopa equivalent dose.

**Results:**

In both freezers and nonfreezers, both RAS-supported multimodal training and regular training significantly improved the Mini-BESTest scores compared with baseline scores and with the control group scores. The improvement was larger for RAS-supported training compared with regular training, for both freezers and nonfreezers. Only the RAS-supported training group retained the improvements compared with baseline measurements at 6-month follow-up, and this was true for both freezers and nonfreezers.

**Conclusions:**

RAS-supported multimodal training is effective in improving balance performance in both freezers and nonfreezers.

**Impact:**

Until this study, it was unknown whether both freezers and nonfreezers could benefit from multimodal balance training. With this information, clinicians who work with people with PD will be better able to apply personalized gait rehabilitation.

**Lay Summary:**

Adding rhythmic auditory stimuli (RAS) to balance training is beneficial for both freezers and nonfreezers, at least in persons with mild to moderate disease stages. This RAS-supported multimodal training has good potential for a wider clinical implementation with good long-term effects.

Nonpharmacological interventions are an important element of the management of gait and balance impairments in people with Parkinson disease (PD).^1^^,^^2^ Particularly, physical therapy can improve gait and balance.^3–7^ A recent prospective randomized clinical trial studied the efficacy of adding rhythmic auditory stimuli (RAS) to standard physical therapy to improve gait and balance (RAS-supported multimodal balance training).^8^ In this study, the effects were compared with both regular multimodal balance training (but without RAS) and a control group who received an educational program. Both RAS-supported multimodal training and regular training improved balance and gait performance after 5 weeks of training, compared with controls. The effects were larger for the RAS-supported training group than for regular training. Only the RAS-supported training group retained the effects at long-term follow-up (6 months).

These results are promising, but to reach the full potential of nonpharmacological interventions, individually tailored treatment is needed.^9^ To this end, clinicians must know which subgroup of patients with PD benefits most from an intervention. Previous subgroup analyses showed that patients with PD who had freezing of gait can respond differently to nonpharmacological interventions compared with nonfreezers.^10^ For example, freezers perform better with closed-loop cueing strategies compared with open-loop strategies,^10^ but this difference was not found for nonfreezers. Moreover, the optimal cueing frequency for freezers is somewhat lower than the baseline step frequency, whereas nonfreezers perform optimally with a cueing frequency of 10% above their baseline stepping frequency.^11^^,^^12^ Additionally, freezers have less retention of dual-task training compared with nonfreezers.^11^ However, a recent secondary analysis on the V-TIME study involving a treadmill intervention did not find a difference between freezers and nonfreezers.^13^ A possible explanation for this lack of difference could be the fact that freezers and nonfreezers did not differ with respect to cognitive impairments.

Here, we present a secondary analysis of the Multimodal Balance Training trial,^8^ evaluating whether RAS-supported multimodal balance training is effective in both freezers and nonfreezers. Because our intervention specifically focused on balance, we expected that both freezers and nonfreezers would improve on the primary outcome measures (Mini-BESTest), because balance impairments occur in both subgroups. Second, we aimed to evaluate whether possible treatment effects would be retained over time, in both freezers and nonfreezers.

## Methods

### Study Design and Participants

The design and outcomes of the RAS-supported multimodal balance trial have been published elsewhere.^8^^,^^14^ In summary, we performed a single-blind, randomized, controlled trial between July 2015 and May 2017 at the Movement Disorders Clinic of the University of São Paulo Faculty Medicine Clinics Hospital. The study was approved by the local ethical committee and participants signed an informed consent form before participation. The protocol was registered at clinicaltrials.gov (NCT02488265).

After screening for eligibility, subjects were randomly assigned (1:1:1) into either 2 experimental groups (RAS-supported multimodal balance training, regular multimodal balance training) or 1 control intervention group (educational program). Randomization was performed by an independent study collaborator before baseline assessments by a computerized block randomization procedure (block size 6) using stratification for disease stage (Hoehn & Yahr stage). Inclusion criteria were: (1) diagnosis of PD according to the UK Brain Bank criteria^15^; (2) Hoehn & Yahr stage 1–3^16^; (3) history of falls in the past year; and (4) Mini Mental Status Examination score 24 or greater.^17^ No other physical therapy interventions or complementary exercises were allowed during the study. Exclusion criteria were Hoehn & Yahr stage greater than 3, dementia or other severe cognitive impairment (Mini Mental Status Examination score less than 24), history of other neurological disorders, orthopedic or cardiopulmonary disease; no stable deep brain stimulation (DBS)-setting; excessive low back pain; unable to attend or adhere to the training schedule; or problems with transportation. We included all freezers and nonfreezers who fulfilled the inclusion criteria. More freezers were excluded (n = 9) because of cognitive impairments compared with nonfreezers (n = 4).

We categorized patients as freezers or nonfreezers based on either self-report (if freezing was present subjectively, as indicated in the New Freezing of Gait Questionnaire [NFOG-Q]^18^) or when patients manifested freezing of gait during the Rapid Turns Test.^19^ Measurements were performed by a blinded assessor at 4 time points: baseline (ie, 14 days prior to training); directly after the last training (5 weeks); at 1-month follow-up; and at 6-months follow-up. Both assessors and patients were instructed not to talk about the allocation. All participants were tested while taking their usual Parkinson medication (ON-medication state), at least 1 h after ingestion of their regular dose of levodopa.

### Interventions

The intervention was delivered by trained physical therapists. Both training groups received multimodal balance training; 1 training group received all exercises combined with RAS provided by a metronome (RAS-supported), whereas the other training group received regular multimodal balance training without RAS. Both intervention groups also received gait training with visual cues (because this is part of routine physical therapy care), but RAS to maximize the balance exercises were only added in the RAS-supported training group. Training in both intervention groups involved 40 balance and gait exercises, provided during 10 sessions of 45 minutes (2 sessions per week over a 5-week period). We chose exercises that are common in routine clinical treatment of gait and balance. No modification of the intervention protocol occurred in other to benefit freezers.

The control group received no functional balance or gait training, but was offered a general education program about PD, falls prevention, and self-care, which also involved 10 sessions of 45 minutes.^8^ Details of the exercises, training progression, and intensity are described elsewhere^8^ and also can be found in Table 1. Table 1 shows an overview of the training program contents and prescription of the RAS-supported multimodal balance training (supported by RAS) and regular multimodal training (without RAS)^8^ and control intervention (educational program).^4^^,^^8^^,^^20^^,^^21^ Please note that these interventions were exactly the same for freezers and nonfreezers.

**Table 1:** Training Program Contents and Prescription of the Rhythmical Auditory Stimuli (RAS)-Supported Multimodal Balance Training, Regular Multimodal Training (Without RAS), and Control Intervention (Educational Program)

	**Frequency/Intensity**	**Duration/Dose**	**Progression/Dose**
**Intervention**	**2x/wk (during 5 wk)**	**45 min: Warm-up, 5 min; Main Part, 30 min; Cool Down, 10 min**	**Personalized**
General content	Both interventions (RAS-supported multimodal balance training and regular multimodal training) consisted of 3 parts: balance, gait, and functional movements^8^ The auditory rhythmical cues in the RAS-group were delivered in an open-loop fashion (throughout the whole duration of the exercises) by a metronome at 50, 80, 100, 120 or 140 bpm (beats per min), according to the exercises. When the patient was unable to perform the movement safely or with enough quality, the rhythm was personalized, and the patient was instructed to use double the time to execute the movements (eg, 100 bpm instead of 50 bpm)	**Week 1**—each exercise was explained and demonstrated by the physical therapist; patients were encouraged to pay attention on the most difficult aspects of movement execution. They performed 5 repetitions of each movement/exercise (5 RM)	**Weeks 2 and 3**—the subjects performed a series of up to 10 RM and in the third week, 2 series of up to 10 RM (20 RM in total) **Weeks 4 and 5**—if the subjects were able to execute 20 RM, they were instructed to progressively increase or decrease the movement speed depending on the exercise (thereby making the exercise more difficult)
Exercises involve 5 elements of posture, gait, and balance:	(a) Sensory integration (eg, walking tasks on varying surfaces) (b) Anticipatory postural adjustments (eg, voluntary arm, leg, and trunk movements, postural transitions, and multidirectional stepping) (c) Compensatory postural adjustments and motor agility (eg, interlimb coordination under varying gait conditions and quick shifts of movement during predictable and unpredictable conditions) (d) Performance at stability limits (eg, controlled leaning tasks performed while standing with varying bases of support, stimulating weight shifts in multiple directions and turning) (e) Use of attentional strategies (maintenance of attention to the gait and balance task)		
Balance	Anticipatory postural adjustments (postural transitions, and multidirectional stepping, emphasizing movement velocity and amplitude) Compensatory postural adjustments (quick shifts of postures during predictable and unpredictable conditions) Performance at stability limits (controlled leaning tasks performed while standing with varying bases of support, stimulating weight shifts in multiple directions and turning) Attentional strategies (maintenance of attention during all exercises)	Patients who did not show a good performance in Week 1 received additional instructions from a physical therapist in terms of how to perform the exercises correctly In the RAS-supported group, patients were instructed to perform the movements slowly (twice the beat of the metronome) (see example in supplementary video^8^).	Decreasing and increasing the base of support Increasing and decreasing movement velocity and amplitude Progress—not holding on wall, bar, or chair
Gait	Motor agility (eg, interlimb coordination under varying gait conditions and quick shifts of movement characteristic during predictable and unpredictable conditions) Performance at stability limits (controlled leaning tasks performed while walking with varying bases of support, in multiple directions and turning) Attentional strategies (maintenance of attention during all exercises)	Focusing on big steps, and visual cues In the RAS-supported group, patients were instructed to perform the movements on the beat of the metronome (see example in supplementary video^8^)	Increase environmental demands: uneven and narrow surfaces, spaces, doorways, varying bases of support, in multiple directions and turning
Functional movements	Performance at stability limits (controlled leaning tasks performed while standing with varying bases of support, stimulating weight shifts in multiple directions and turning) Posture core stabilization Attentional strategies (maintenance of attention during all exercises)	Focusing attention on the most difficult part of the movement In the RAS-supported group, patients were instructed to perform the movements slowly (twice the beat of the metronome)	Increase coordination demands; sit-to-stand, start-stop Increase or decrease the speed
Materials: Chairs, balls (65 cm, 55 cm, 30 cm); rubber bands (120 cm—medium density); sticks (100 cm), foams: 160 x 23 x 6 cm; 50 x 41 x 6 cm; 100 x 41 x 6 cm; 185 x 100 x 1.5 cm); bar, colored adhesive tapes; MA-1 KORG metronome.			
Educational program	Contents of components were distributed in lectures, video sessions, general orientations about Parkinson disease and discussions^4^^,^^20^	45 min The educational program was delivered in Portuguese (because the study was conducted in Brazil and all participants spoke Portuguese)^21^	
Lectures	**Week 1.** Parkinson disease—epidemiology, pathology and diagnosis **Week 2.** Treatment pharmacological, neurosurgery, and rehabilitation (general information) **Week 3.** Quality of life and self-management **Week 4.** Patient-centered care. How to be involved **Week 5.** Falls prevention	5 min—Welcome 30 min—Lecture 10 min—Discussion	
Video session	**Week 6**. Advocating for Parkinson disease. Videos of famous people with Parkinson disease **Week 7.** Discussion about lectures and videos, sessions (hot topics chosen by the group)	5 min—Welcome 30 min—Video 10 min—Discussion	
Orientations and discussions	**Week 8.** Make a list of your PD problems and try guiding us to help you **Week 9.** What people with Parkinson disease should do and are able to do. Making your plans for the future **Week 10.** Doubts (question and answers about all educational program topics)	5 min—Welcome 30 min—Orientations 10 min—Discussion	

### Outcomes

This secondary subgroup analysis for freezers and nonfreezers included the Mini-BESTest (MBEST) as primary outcome^22^; this test has a maximum score of 28 points for 14 items that are each scored from 0 to 2.^23^ Secondary outcomes were Timed Up and Go Test (TUG)^24^ and the NFOG-Q.^18^

### Statistical Analysis

Statistical analysis was performed according to the intention-to-treat principle using SPSS version 22 (IBM Corp, Armonk, New York, USA). Linear mixed models were used for all outcomes. The primary end point was the MBEST directly postintervention. We used treatment (RAS-supported training vs regular training vs controls), visit (immediately postintervention, 1-month follow-up, and 6 months follow-up), subgroup (freezer vs nonfreezers), and the 2- and 3-fold interactions between visit, treatment group, and subgroups (freezers/nonfreezers) as fixed factors. To adjust for baseline imbalances, the model was adjusted for baseline MBEST, Unified Parkinson’s Disease Rating Scale parts 2 and 3, and levodopa equivalent daily dose. Patient was included as a random factor. In separate linear mixed models, we modeled the outcomes of the TUG and NFOG-Q tests. These models were built in the same way as we modeled the MBEST test, but for each of these models we additionally adjusted for the baseline measure of the outcome variable. The descriptive statistics included means, SDs, and 95% CIs.

### Role of the Funding Source

The funder played no role in the design, conduct, or reporting of this study.

## Results

Baseline characteristics of the 154 participants (freezers [n = 59], nonfreezers [n = 95]) are presented in Table 2. Post hoc tests revealed that freezers in the control group had a higher Unified Parkinson’s Disease Rating Scale part 2 score compared with freezers in both intervention groups (*P* < .05). All other baseline parameters did not differ between the groups, both for freezers and nonfreezers (Tab. 2).

**Table 2:** Subgroup Participants’ Characteristics at Baseline Visit^*a*^

	**Freezers (n = 59)**			**Nonfreezers (n = 95)**		
**Variable**	**RAS-Supported (n = 19)**	**Regular (n = 18)**	**Control (n = 22)**	**RAS-Supported (n = 36)**	**Regular (n = 34)**	**Control (n = 25)**
Age, y, mean [SD]	74 [8]	63 [13]	76 [7]	72 [9]	68 [12]	70 [10]
Sex, men, n (%)	10 (55.6)	9 (50)	18 (81.8)	16 (42.9)	22 (78.6)	11 (44)
H&Y grade, n (%)						
I	0 (0.0)	0 (0.0)	0 (0.0)	12 (34.8)	14 (39.4)	7 (26)
II	2 (11)	3 (17.6)	4 (18.2)	11 (32.9)	10 (30.3)	6 (24)
III	16 (88.9)	14 (82.4)	18 (72.7)	10 (32.3)	10 (30.3)	12 (50)
Disease duration, y, median (IQT)	8 (3–12)	10 (4–16)	10 (3–15)	3 (2–8)	5 (2–7)	7 (1–10)
LEDD, mg/d, mean [SD]	902 [514]	889 [493]	913 [457]	563 [430]	557[359]	611 [324]
MMSE, score, mean [SD]	26 [2]	25 [2]	24 [2]	27 [2]	25 [2]	26 [2]
MoCA, score, mean [SD]	25 [3]	24 [2]	23 [2]	26 [3]	24 [3]	25 [3]
UPDRS 2, ADL score, mean [SD]	19 [7]	20 [8]	23 [6]	10 [6]	12 [6]	12 [6]
UPDRS 3, motor score, mean [SD]	20 [7]	23 [10]	24 [7]	13 [6]	15 [6]	17 [7]
Mini BESTest, mean (SE)	11.0 (1.5)	9.9 (1.6)	11.8 (1.4)	16.8 (1.1)	21.4 (1.1)	18.1 (1.4)
New Freezing of Gait Questionnaire, mean (SE)	14.2 (1.3)	21.0 (1.4)	15.3 (1.2)	0 (0)	0 (0)	0 (0)
Timed Up and Go Test, mean (SE)	33.4 (3.5)	28.5 (3.6)	25.8 (3.2)	18.4 (2.5)	15.9 (2.7)	18.9 (3.1)

^*a*^ADL = Activities of daily living; IQT = interquartile range; LEDD = levodopa equivalent daily dose; MMSE = Mini Mental Status Examination; MoCA = Montreal Cognitive Assessment; UPDRS = Unified Parkinson’s Disease Rating Scale; SD = standard deviation; SE = standard error

### Balance

The Figure shows the results for our primary outcome, the MBEST. Immediately posttreatment, both freezers and nonfreezers in the RAS-supported and regular training groups improved their MBEST score compared with baseline (Tab. 4) and compared with participants in the control group (Tab. 3). Moreover, RAS-supported training afforded significantly larger improvement than regular training, both for freezers and nonfreezers (Tab. 3). Compared with the postintervention measurement, freezers in both the RAS-supported group and regular training group did not significantly change in their MBEST scores at 1-month follow-up and improvements were therefore retained. Compared with the postintervention measurements, scores did deteriorate at 6 months in both intervention groups (Tab. 4). However, compared with the baseline measurement, freezers in the RAS-supported group still had MBEST improvements at 6-months follow-up, and improvements in freezers in the RAS-supported group were therefore retained compared with baseline measurements. Compared with the postintervention measurement, nonfreezers in both the RAS-supported and regular training groups did not change MBEST scores at 1-month follow up, and improvements were therefore retained. When looking at the postintervention measurements, improvements were not retained at 6-months follow-up in either intervention group, but compared with the baseline measurement, nonfreezers in the RAS-supported group still had MBEST improvements at 6-months follow.

**Table 3:** Observed Mean Values and Estimated Between-Group Differences in Improvement From Baseline and 95% CIs for Balance and Gait Outcomes for Freezers and Nonfreezers^*a*^

**Groups** *^b^*		**RAS-Supported**	**Regular**	**Control**	**RAS-Supported vs Regular**	**RAS-Supported vs Control**	**Regular vs Control**
Mini BESTest*^c^*							
Freezers	Baseline	11.1 (7.8 to 14.3)	11.7 (8.2 to 15.2)	11.2 (7.8 to 14.5)			
	Postintervention	18.4 (15.3 to 21.5)	14.7 (10.8 to 18.4)	11.2 (7.9 to 14.4)	4.0 (2.0 to 6.5) *P* < .001	7.3 (5.0 to 9.0) *P* < .001	3.4 (0.7 to 5.0) *P* = .011
	1-mo follow-up	17.6 (14.0 to 21.2)	13.9 (9.9 to 17.9)	11.2 (7.8 to 14.5)	4.1 (1.9 to 6.4) *P* < .001	6.5 (4.2 to 8.8) *P* < .001	2.3 (−0.1 to 4.7) *P* = .051
	6-mo follow-up	15.8 (12.0 to 19.5)	12.9 (8.9 to 16.8)	11.0 (11.3 to 20.3)	3.2 (1.0 to 5.5) *P* = .004	4.5 (2.2 to 6.9) *P* < .001	1.3 (−1.0 to 3.6) *P* = .278
Nonfreezers	Baseline	16.7 (14.5 to 18.8)	20.7 (17.8 to 23.6)	17.0 (12.4 to 21.5)			
	Postintervention	23.0 (21.0 to 25.0)	23.3 (20.6 to 26.0)	16.2 (12.3 to 21.1)	3.1 (1.4 to 4.8) *P* < .001	6.5 (4.7 to 8.4) *P* < .001	3.4 (1.4 to 65.4) *P* = .001
	1-mo follow-up	22.4 (20.0 to 24.4)	21.6 (18.4 to 24.3)	17.2 (11.9 to 20.4)	4.0 (2.3 to 5.7) *P* < .001	6.3 (4.4 to 8.1) *P* < .001	2.2 (0.2 to 4.2) *P* = .030
	6-mo follow-up	20.8 (18.7 to 23.0)	20.0 (17.5 to 22.6)	15.8 (11.3 to 20.3)	4.2 (2.4 to 5.9) *P* < .001	5.2 (3.4 to 7.1) *P* < .001	1.0 (−0.9 to 3.0) *P* = .297
New Freezing of Gait Questionnaire*^d^*							
Freezers	Baseline	15.2 (12.2 to 18.2)	20.4 (16.4 to 24.5)	15.1 (12.1 to 18.2)			
	Postintervention	12.2 (9.2 to 14.9)	17.7 (14.7 to 20.6)	15.1 (12.0 to 18.1)	0.1 (−0.5 to 0.2) *P* = .389	−2.1 (−2.7 to −1.6) *P* < .001	−2.0 (−2.4 to −1.5) *P* < .001
	1-mo follow-up	12.9 (9.4 to 15.16)	18.4 (15.6 to 21.2)	16 (12.8 to 19.1)	−0.6 (−1.0 to −0.2) *P* = .002	−1.0 (−1.6 to −0.4) *P* = .001	−0.4 (−0.8 to 0.0) *P* = .067
	6-mo follow-up	14.2 (11.2 to 17.3)	18.8 (16.3 to 21.4)	16 (12.8 to 19.1)	−1.0 (−1.4 to −0.5) *P* < .001	−1.0 (−1.6 to −0.3) *P* = .002	−4.5 (−0.4 to 0.4) *P* = 1.000
Nonfreezers	Baseline	0.0 (0.0 to 0.0)	0.0 (0.0 to 0.0)	0.0 (0.0 to 0.0)			
	Postintervention	2.3 (0.3 to 4.5)	1.9 (0.3 to 3.4)	3.7 (0.9 to 6.4)	−0.0 (−0.3 to 0.2) *P* = .839	−0.4 (−0.8 to −0.0) *P* = .049	−0.3 (−0.6 to −0.0) *P* = .009
	1-mo follow-up	2.3 (0.3 to 4.4)	2.4 (0.3 to 3.7)	3.6 (0.8 to 6.5)	−0.1 (−0.4 to 0.2) *P* = .477	−0.1 (−0.5 to 0.3) *P* = .618	−0.0 (−0.4 to 0.3) *P* = .999
	6-mo follow-up	2.7 (0.4 to 5.1)	2.1 (0.4 to 3.8)	3.6 (0.8 to 6.4)	0.0 (−0.3 to 0.4) *P* = .774	0.0 (−0.5 to 0.6) *P* = .838	0.1 (−4.0 to 4.2) *P* = .947
Timed Up and Go Test*^e^*							
Freezers	Baseline	33.5 (23.9 to 43.1)	27.0 (16.0 to 38.0)	26.2 (17.0 to 35.4)			
	Postintervention	21.9 (14.3 to 29.5)	18.0 (10.3 to 25.8)	27.1 (16.9 to 37.2)	0.8 (−0.7 to 1.7) *P* = .071	2.7 (1.4 to 3.9) *P* < .001	1.8 (0.9 to 2.7) *P* < .001
	1-mo follow-up	21.4 (13.5 to 29.4)	20.4 (12.7 to 28.0)	28.3 (17.3 to 39.2)	0.7 (−0.2 to 1.6) *P* = .130	1.7 (0.4 to 3.0) *P* = .008	1.0 (0.0 to 1.9) *P* = .039
	6-mo follow-up	23.7 (15.5 to 32.0)	21.7 (13.5 to 29.0)	29.0 (18.3 to 39.6)	0.4 (−0.9 to 0.9) *P* = 1.000	1.7 (0.4 to 3.0) *P* = .008	1.0 (0.0 to 1.9) *P* = .039
Nonfreezers	Baseline	18.9 (14.9 to 22.8)	14.6 (11.6 to 17.1)	20.5 (12.5 to 28.5)			
	Postintervention	14.6 (12.1 to 17.3)	12.0 (9.7 to 12.3)	21.1 (12.8 to 29.5)	0.7 (0.1 to 1.4) *P* = .014	2.1 (1.3 to 3.0) *P* < .001	1.3 (0.7 to 2.0) *P* < .001
	1-mo follow-up	13.5 (11.5 to 15.4)	12.8 (10.6 to 14.9)	20.9 (12.7 29.1)	1.7 (0.9 to 2.4) *P* < .001	3.2 (2.2 to 4.2) *P* < .001	1.5 (0.7 to 2.2) *P* < .001
	6-mo follow-up	14.6 (12.1 to 17.1)	14.0 (11.9 to 16.7)	22.8 (12.7 to 33.0)	0.5 (−0.3 to 1.4) *P* = .244	0.8 (−0.3 to 2.0) *P* = .151	0.3 (−0.5 to 1.2) *P* = .437

^*^a^*^LEDD = levodopa equivalent daily dose; MMSE = Mini Mental Status Examination; NFOG = New Freezing of Gait Questionnaire; RAS = rhythmical auditory stimuli; UPDRS = Unified Parkinson’s Disease Rating Scale.

^*^b^*^Group: Multimodal balance training supported by RAS, multimodal balance training without RAS (regular), control intervention group (control).

^*^c^*^Mini BEST: Between-group differences (95% CI) in improvement from baseline; adjusted for baseline Mini BESTest, baseline LEDD, and baseline UPDRS 2 and 3. Mini BESTest: 14 items, total 28 of points, scored 0–2 (higher score = better balance).

^*^d^*^New Freezing of Gait Questionnaire (NFOG): Adjusted NFOG mean difference (95% CI) from baseline. Adjusted for NFOG baseline, baseline LEDD, baseline UPDRS 2 and 3.

^*^e^*^Timed Up and Go Test (TUG): Adjusted TUG mean difference (95% CI) from baseline. Adjusted for baseline TUG, baseline LEDD, baseline UPDRS 2 and 3. TUG was measured in time in seconds with a range 5–60 s. A lower score means better mobility.

**Table 4:** Estimated Differences Between Baseline and Postintervention Measures With 95% CIs for Primary and Secondary Outcomes, for Freezers and Nonfreezers*^a^*

		**Difference Compared With Baseline Measurement**			**Difference Compared With Postintervention Measurement**		
**Groups** *^b^*		**RAS-Supported**	**Regular**	**Control**	**RAS-Supported**	**Regular**	**Control**
Mini BESTest*^c^*							
Freezers	Postintervention	6.8 (5.2 to 8.3)	2.5 (0.9 to 4.2)	−0.5 (−2.3 to 1.2)			
	1-mo follow-up	5.9 (4.4 to 7.5)	1.8 (0.1 to 3.4)	−0.5 (−2.3 to 1.2)	−0. 8 (−0.7 to 0.0)	−0.7 (−1.6 to 0.2)	0.0 (−0.9 to 0.9)
	6-mo follow-up	4.1 (2.5 to 5.6)	0.8 (−0.8 to 2.4)	−0.4 (−2.3 to 1.3)	−2.7 (−3.9 to −1.5)	−1.7 (−3.0 to −0.4)	0.0 (−1.2 to 1.4)
Nonfreezers	Postintervention	6.4 (5.2 to 7.5)	3.2 (1.9 to 4.6)	−0.1 (−1.6 to 1.3)			
	1-mo follow-up	5.6 (4.4 to 6.7)	1.5 (0.2 to 2.8)	−0.6 (−2.2 to 0.8)	−0.7 (−1.4 to −0.1)	−1.7 (−2.4 to −0.9)	−0.5 (−1.4 to 0.3)
	6-mo follow-up	4.2 (3.1 to 5.3)	0.0 (−1.2 to 1.3)	−1.0 (−2.5 to 0.5)	−2.1 (−3.0 to −1.3)	−3.2(−4.2 to −2.2)	−0.8 (−2.0 to 0.3)
New Freezing of Gait Questionnaire*^d^*							
Freezers	Postintervention	−1.5 (−3.3 to 0.2)	0.0 (−2.4 to 2.5)	0.5 (−1.8 to 2.8)			
	1-mo follow-up	−1.4 (−3.1 to 0.3)	0.7 (−1.8 to 3.2)	1.5 (−0.8 to 3.8)	7,7 (4.1 to 11.2)	8.2 (4.7 to 11.7)	7.0 (3.4 to 10.5)
	6-mo follow-up	0.0 (−1.7 to 1.7)	1.1 (−1.4 to 3.6)	1.5 (−0.8 to 3.8)	3.6 (−0.0 to 7.3)	4.4 (0.7 to 8.2)	2.8 (−0.8 to 6.5)
Nonfreezers	Postintervention	0.9 (−0.6 to 2.4)	−0.2 (−1.9 to 1.4)	2.1 (0.1 to 4.1)			
	1-mo follow-up	0.9 (−0.6 to 2.4)	0.0 (−1.7 to 1.6)	2.1 (0.1 to 4.1)	−1.7 (−4.6 to 1.2)	−1.6 (−4.6 to 1.3)	−2.0 (−5.0 to 0.9)
	6-mo follow-up	1.3 (−0.1 to 2.9)	0.0 (−1.7 to 1.6)	2.1 (0.1 to 4.1)	0.3 (−2.8 to 3.6)	0.3 (−2.9 to 3.5)	−0.1 (−3.3 to 3.1)
Timed Up and Go Test*^e^*							
Freezers	Postintervention	−8.2 (−11.4 to −5.1)	−9.2 (−12.9 to −5.5)	0.7 (−3.1 to 4.6)			
	1-mo follow-up	−9.3 (−12.4 to −6.2)	−6.9 (−10.6 to −3.2)	1.8 (−2.0 to 5.7)	1.6 (−1.1 to 4.3)	2.1 (−0.5 to 4.8)	1.1 (−1.5 to 3.8)
	6-mo follow-up	−7.3 (−10.5 to −4.2)	−6.0 (−9.7 to −2.3)	2.2 (−1.6 to 6.1)	2.0 (−0.5 to 4.7)	2.9 (0.2 to 5.5)	1.5 (−1.1 to 4.1)
Nonfreezers	Postintervention	−4.3 (−7.2 to −1.6)	−3.9 (−6.8 to −1.0)	0.8 (−2.6 to 4.3)			
	1-mo follow-up	−5.1 (−7.8 to −2.4)	−3.2 (−6.1 to −0.2)	0.7 (−2.7 to 4.2)	−1.1 (−3.2 to 0.9)	−1.0 (−3.1 to 1.0)	−1.4 (−3.5 to 0.6)
	6-mo follow-up	−3.9 (−6.6 to −1.2)	−2.0 (−4.9 to 0.9)	2.8 (−0.6 to 6.3)	1.2 (−1.0 to 3.5)	1.1 (−1.0 to 3.4)	0.7 (−1.5 to 3.0)

^*^a^*^LEDD = levodopa equivalent daily dose; MMSE = Mini Mental Status Examination; RAS = rhythmic auditory stimuli; UPDRS = Unified Parkinson’s Disease Rating Scale.

^*^b^*^Group: Multimodal balance training supported by RAS (RAS-supported), multimodal balance training without RAS (regular), control intervention group (control).

^*^c^*^Mini BEST: between-groups differences (95% CI) in improvement from baseline; adjusted for baseline MBEST, baseline LEDD, and baseline UPDRS 2 and 3. Mini BESTest—14 items, total 28 of points, scored 0-2 (higher score better balance).

^*^d^*^New Freezing of Gait Questionnaire (NFOG): Adjusted NFOG mean difference (95% CI) from baseline. Adjusted for NFOG baseline, baseline LEDD, and baseline UPDRS 2 and 3.

^*^e^*^Timed Up and Go Test (TUG): TUG—adjusted TUG mean difference (95% CI) from baseline. Adjusted for baseline TUG, baseline LEDD, baseline UPDRS 2 and 3. TUG was measured in time in seconds with a range from 5 to 60 s. A lower score means better mobility.

### Freezing of Gait

Freezers in both training groups did not significantly improve their NFOG-Q scores directly posttreatment or at follow-up (Tab. 4). However, at 1-month and 6-months follow-up freezers in the RAS-supported group showed a better result than freezers in the regular training group or control group (Tab. 3 and Figure), because NFOG-Q scores deteriorated in the latter 2 groups (Tab. 4).

**Figure:**
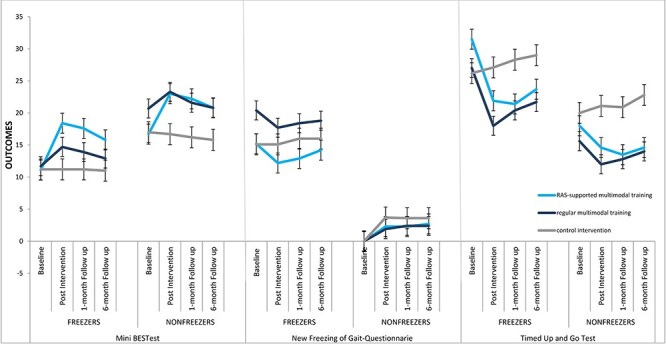
Mini-BESTest, New Freezing of Gait Questionnaire, and Timed Up and Go Test at each test visit. The blue line represents multimodal balance training supported by rhythmical auditory stimuli (RAS-supported), the dark-blue line represents multimodal balance training without RAS (regular), and the gray line represents the control intervention group. Error bars represent the 95% CIs.

### Functional Mobility: Freezers versus Nonfreezers

Freezers and nonfreezers in both training groups improved their TUG score immediately posttreatment compared with baseline measurement (Tab. 4) and compared with participants in the control group (Figure). For freezers, no significant differences were found between RAS-supported and regular training, whereas nonfreezers improved more with RAS-supported training than with regular training. At 1-month follow-up, TUG scores in both freezers and nonfreezers did not differ compared with the postintervention measurements in both intervention groups, and improvements were therefore maintained. At 6-months follow-up, TUG scores did not differ compared with postintervention measurements in freezers in the RAS-supported training group, but did increase in freezers in the regular training group (although improvement was retained compared with the baseline measurement [Tab. 4]). In nonfreezers, TUG scores did not differ compared with postintervention measurements in either training group at 6-months follow-up, and improvements were therefore sustained (Tabs. 3 and 4).

## Discussion

In this secondary analysis, we evaluated whether multimodal balance training supported by RAS is effective in both freezers and nonfreezers. In line with our hypothesis, both freezers and nonfreezers in the RAS-supported and regular training groups significantly improved their MBEST score immediately posttreatment compared with baseline scores and compared with controls. This improvement was larger for RAS-supported training than for regular training. Moreover, only participants receiving RAS-supported training retained the improvements at 6-months follow-up (compared with baseline measurements), and this was true for both freezers and nonfreezers. These findings indicate that both freezers and nonfreezers respond largely similarly compared to the overall group reported earlier in our previous study.^8^ When aiming to improve balance performance in persons with PD, adding external auditory cues to exercises should therefore be considered, irrespective of whether patients freeze or not.

With respect to gait, we report 2 new findings. First, RAS-supported multimodal balance training was not effective in reducing freezing of gait, as measured with the N-FOGQ. This is surprising, because there is a large body of evidence showing beneficial effects of external auditory cueing on gait performance in persons with PD.^7^^,^^10^^,^^25^^,^^26^ However, our training program focused primarily on balance, and external cues were not used after the training in daily life situations, which might explain these findings. Additionally, it becomes increasingly clear that measuring the effect of a training intervention on freezing of gait using the NFOG-Q has limitations.^27^^,^^28^ A recent study reported that the NFOG-Q has a minimal detectable change of 9.9 points (relative minimal detectable change of 35.5%).^28^ Future studies are therefore needed to quantify the effects using additional measures that could capture clinical relevance, including, for example, objective measures (eg, using wearable sensors), ideally in the home setting and over longer time frames.^27^ Future studies could also evaluate whether RAS-supported multimodal training can help to prevent occurrence of freezing in nonfreezers, thereby facilitating delivery of a wide range of physical therapy interventions focusing on improving gait or balance, or delivery of exercise training.

A second important finding is that functional mobility (TUG) improved in both freezers and nonfreezers. The degree of improvement exceeded the minimal clinically relevant difference of 3.5 seconds.^29^ Among freezers, we found no differences between both active interventions on the TUG outcomes, which is consistent with our primary analysis.^8^ However, among nonfreezers, the RAS-supported training group improved significantly more than the regular training group when measured directly after the intervention. This is surprising, because one would expect that particularly freezers would benefit from additional cueing. This lack of greater improvement in freezers might be due to a power problem, because the degree of improvement was larger following RAS-supported training compared with regular training.

In contrast to several other intervention studies,^10–12^ multimodal training (with and without auditory cues) was effective in both freezers and nonfreezers. Differential effects between freezers and nonfreezers could arise because of differences in disease severity (freezers are often more severely affected than nonfreezers) or the presence of cognitive impairments (cognitive impairments are more frequently seen in freezers than in nonfreezers). In our study, disease severity was longer in freezers, but scores of cognitive tests (Tab. 2) were comparable between freezers and nonfreezers (because cognitive impairment was an exclusion criterion). The lack of differences in cognitive status might explain why we did not find differences between both subgroups. This is in line with a recent study from Bekkers and colleagues,^13^ which also found no difference between freezers and nonfreezers when evaluating a treadmill intervention. In this study, freezers and nonfreezers did not differ with respect to cognitive impairments. Apparently, cognitive status could be an important determinant of the efficacy of physical therapy and possibly other comparable nonpharmacological interventions. This aspect deserves further study in future work.

Our study is not without limitations. First, we only included patients without cognitive impairments and who were in mild to moderate disease stages. Future studies should also investigate the effects of our intervention in persons with PD with more pronounced cognitive impairments, because this could be an important determinant of the efficacy of nonpharmacological interventions such as the RAS-supported intervention tested here. Second, we did not perform a formal power analysis for this subgroup analysis, although we do not expect that the magnitude or direction of the findings would have been different with a larger sample. Third, we cannot make any statements about the possible effects when patients are OFF medication because we purposely tested and trained all patients in an ON-medication state, because this would best reflect the effect of the intervention over and above standard medical management in daily clinical practice, where patients are typically seen and treated during a regular ON state. This, however, means that the primary and secondary outcome scores are a reflection of the patients’ optimal functioning, leading to overall lower scores.^8^ Future studies should examine the effects of RAS-supported multimodal balance training on balance in patients with more advanced stages, cognitive impairments, and also while they are being tested in an OFF-medication state*.*

Taken together, this secondary analysis suggests that adding RAS to balance training is beneficial for both freezers and nonfreezers, at least in persons with mild to moderate disease stages that were included in the present study. Future studies are needed to evaluate the effect of training interventions such as RAS-supported balance training in patients with later disease stages (eg, Hoehn & Yahr stage 4).

## Clinical Trial Registration

The protocol was registered at clinicaltrials.gov (NCT02488265).

## References

[1] Bloem BR , Ypinga JHL, Willis A, et al. Using medical claims analyses to understand interventions for Parkinson patients. J Parkinsons Dis. 2018;8:45–58. 29254108 10.3233/JPD-171277 PMC5836412

[2] Bloem BR , de Vries NM, Ebersbach G. Nonpharmacological treatments for patients with Parkinson's disease. Mov Disord. 2015;30:1504–1520. 26274930 10.1002/mds.26363

[3] Fox SH , Katzenschlager R, Lim SY, et al. International Parkinson and Movement Disorder Society evidence-based medicine review: update on treatments for the motor symptoms of Parkinson's disease. Mov Disord. 2018;33:1248–1266. 29570866 10.1002/mds.27372

[4] Keus S , Munneke M, Graziano M, et al. European physiotherapy guideline for Parkinson’s disease. The Netherlands: ParkinsonNet; 2014.

[5] Schenkman M , Moore CG, Kohrt WM, et al. Effect of high-intensity treadmill exercise on motor symptoms in patients with de novo Parkinson disease: a phase 2 randomized clinical trial. JAMA Neurol. 2018;75:219–226. 29228079 10.1001/jamaneurol.2017.3517 PMC5838616

[6] Conradsson D , Lofgren N, Nero H, et al. The effects of highly challenging balance training in elderly with Parkinson's disease: a randomized controlled trial. *Neurorehabil Neural Repair*. 2015;29:827–836. 25608520 10.1177/1545968314567150 PMC4582836

[7] Mak MK , Wong-Yu IS, Shen X, Chung CL. Long-term effects of exercise and physical therapy in people with Parkinson disease. Nat Rev Neurol. 2017;13:689–703. 29027544 10.1038/nrneurol.2017.128

[8] Capato TTC , de Vries NM, IntHout J, Barbosa ER, Nonnekes J, Bloem BR. Multimodal balance training supported by rhythmical auditory stimuli in Parkinson's disease: a randomized clinical trial. *J Parkinsons Dis*. 2020;10:333–346. 31884492 10.3233/JPD-191752 PMC7029328

[9] Nonnekes J , Nieuwboer A. Towards personalized rehabilitation for gait impairments in Parkinson's disease. *J Parkinsons Dis*. 2018;8:S101–S106. 30584154 10.3233/JPD-181464 PMC6311370

[10] Ginis P , Heremans E, Ferrari A, Bekkers EMJ, Canning CG, Nieuwboer A. External input for gait in people with Parkinson's disease with and without freezing of gait: one size does not fit all. *J Neurol*. 2017;264:1488–1496. 28653213 10.1007/s00415-017-8552-6

[11] Strouwen C , Molenaar E, Munks L, et al. Training dual tasks together or apart in Parkinson's disease: results from the DUALITY trial. Move Disord. 2017;32:1201–1210. 10.1002/mds.27014 28440888

[12] Arias P , Cudeiro J. Effect of rhythmic auditory stimulation on gait in Parkinsonian patients with and without freezing of gait. PLoS One. 2010;5:e9675. 20339591 10.1371/journal.pone.0009675 PMC2842293

[13] Bekkers EMJ , Mirelman A, Alcock L, et al. Do patients with Parkinson's disease with freezing of gait respond differently than those without to treadmill training augmented by virtual reality? Neurorehabil Neural Repair. 2020;34:440–449. 32202203 10.1177/1545968320912756

[14] Capato T , Tornai J, Avila P, Barbosa ER, Piemonte ME. Randomized controlled trial protocol: balance training with rhythmical cues to improve and maintain balance control in Parkinson's disease. *BMC Neurol*. 2015;15:162. 26347052 10.1186/s12883-015-0418-x PMC4561447

[15] Hughes AJ , Daniel SE, Kilford L, Lees AJ. Accuracy of clinical diagnosis of idiopathic Parkinson's disease: a clinico-pathological study of 100 cases. J Neurol Neurosurg Psychiatry. 1992;55:181–184. 1564476 10.1136/jnnp.55.3.181 PMC1014720

[16] Hoehn MM , Yahr MD. Parkinsonism: onset, progression and mortality. *Neurology*. 1967;17:427–442. 6067254 10.1212/wnl.17.5.427

[17] Tombaugh TN , McIntyre NJ. The mini-mental state examination: a comprehensive review. *J Am Geriatr Soc*. 1992;40:922–935. 1512391 10.1111/j.1532-5415.1992.tb01992.x

[18] Nieuwboer A , Rochester L, Herman T, et al. Reliability of the new freezing of gait questionnaire: agreement between patients with Parkinson's disease and their carers. *Gait Posture*. 2009;30:459–463. 19660949 10.1016/j.gaitpost.2009.07.108

[19] Snijders AH , Haaxma CA, Hagen YJ, Munneke M, Bloem BR. Freezer or non-freezer: clinical assessment of freezing of gait. Parkinsonism Relat Disord. 2012;18:149–154. 21968033 10.1016/j.parkreldis.2011.09.006

[20] Capato TTC , Domingos JMM, de Almeida LRS. Versão em Português da Diretriz Europeia de Fisioterapia para a Doença de Parkinson. 1st ed. São Paulo, Brazil: Omni Farma; 2015.

[21] Versão em Português da Diretriz Europeia de Fisioterapia para a Doença de Parkinson. Informações para pessoas com doença de Parkinson [press release]. São Paulo, Brazil: Omni Farma; 2016.

[22] Franchignoni F , Horak F, Godi M, Nardone A, Giordano A. Using psychometric techniques to improve the balance evaluation systems test: the mini-BESTest. J Rehabil Med. 2010;42:323–331. 20461334 10.2340/16501977-0537 PMC3228839

[23] King L , Horak F. On the mini-BESTest: scoring and the reporting of total scores. Phys Ther. 2013;93:571–575. 23547173 10.2522/ptj.2013.93.4.571

[24] Podsiadlo D , Richardson S. The Timed “Up & Go”: a test of basic functional mobility for frail elderly persons. *J Am Geriatr Soc*. 1991;39:142–148. 1991946 10.1111/j.1532-5415.1991.tb01616.x

[25] Lim I , van Wegen E, de Goede C, et al. Effects of external rhythmical cueing on gait in patients with Parkinson's disease: a systematic review. Clin Rehabil. 2005;19:695–713. 16250189 10.1191/0269215505cr906oa

[26] Nieuwboer A , Kwakkel G, Rochester L, et al. Cueing training in the home improves gait-related mobility in Parkinson's disease: the RESCUE trial. *J Neurol Neurosurg Psychiatry*. 2007;78:134–140. 17229744 10.1136/jnnp.200X.097923 PMC2077658

[27] Mancini M , Bloem BR, Horak FB, Lewis SJG, Nieuwboer A, Nonnekes J. Clinical and methodological challenges for assessing freezing of gait: future perspectives. *Move Disord*. 2019;34:783-790. 10.1002/mds.27709 PMC7105152 31046191

[28] Hulzinga F , Nieuwboer A, Dijkstra BW, et al. The new freezing of gait questionnaire: unsuitable as an outcome in clinical trials? *Mov Disord Clin Pract*. 2020;7:199–205. 32071940 10.1002/mdc3.12893 PMC7011794

[29] Huang SL , Hsieh CL, Wu RM, Tai CH, Lin CH, Lu WS. Minimal detectable change of the Timed “Up & Go” test and the dynamic gait index in people with Parkinson disease. Phys Ther. 2011;91:114–121. 20947672 10.2522/ptj.20090126

